# Supplementary material for: A Global Screen for Assembly State Changes of the Mitotic Proteome by SEC-SWATH-MS
Source: Cell Syst. 2020 Feb 26;10(2):133–155.e6. doi: 10.1016/j.cels.2020.01.001 (PMC7042714; doi:10.1016/j.cels.2020.01.001)

Q92625 | ANS1A_HUMAN | ANKS1A ANKS1 KIAA0229 ODIN
Monomer MW [kDa]: 123.108 Monomer expected elution fraction: 38

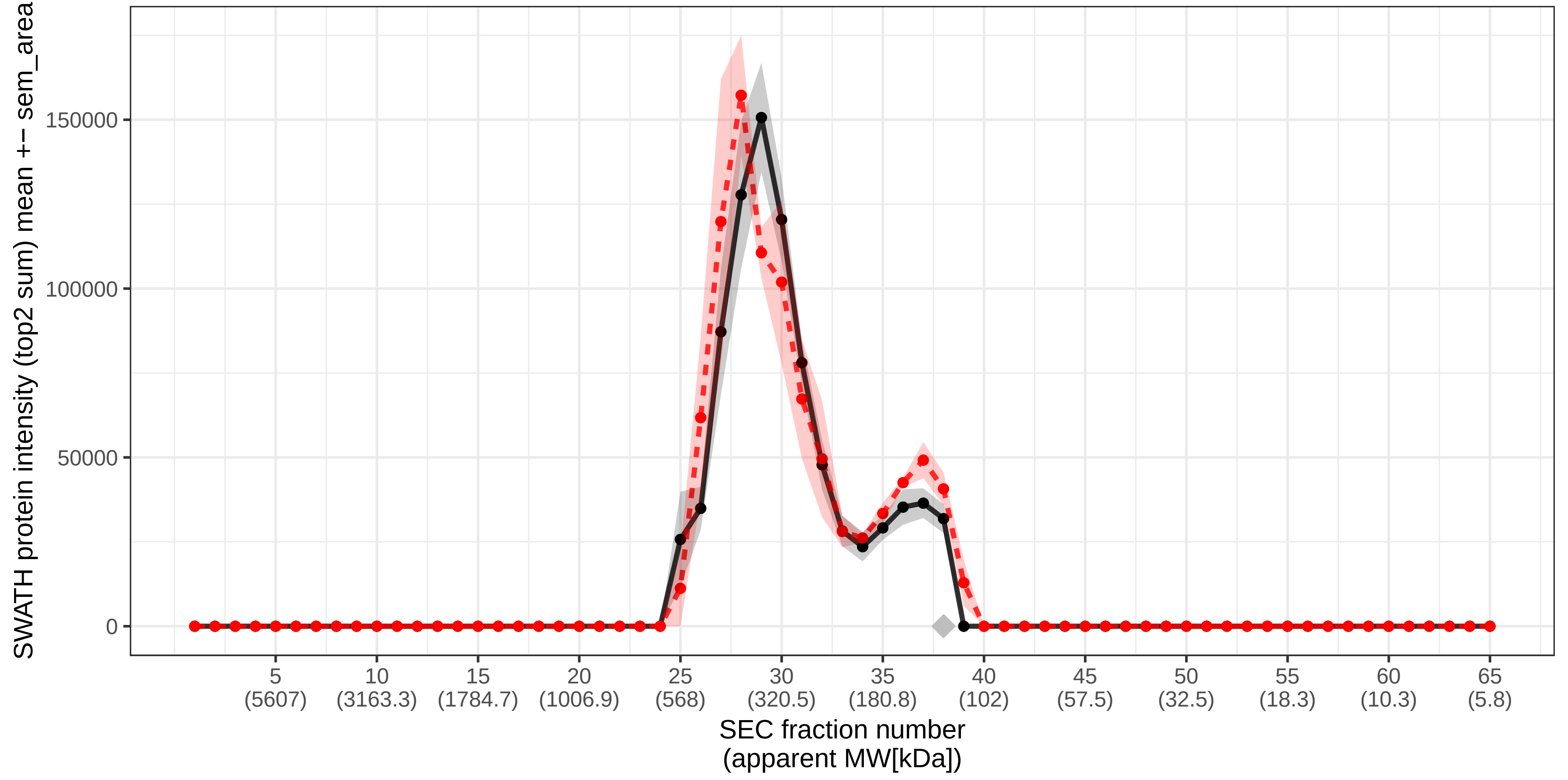

Supplement: Data S1. SEC-SWATH-MS Protein Chromatograms, Related to Figure 1 [file mmc6.zip › SECchrom_Q92625_ANS1A_HUMAN_ANKS1A_ANKS1_KIAA0229_ODIN.pdf]
